# Effects of Neuromuscular Fatigue on Eccentric Strength and Electromechanical Delay of the Knee Flexors: The Role of Training Status

**DOI:** 10.3389/fphys.2019.00782

**Published:** 2019-06-26

**Authors:** Said El-Ashker, Helmi Chaabene, Olaf Prieske, Ashraf Abdelkafy, Mohamed A. Ahmed, Qassim I. Muaidi, Urs Granacher

**Affiliations:** ^1^ Self-Development Department, Deanship of Preparatory Year, Imam Abdulrahman Bin Faisal University, Dammam, Saudi Arabia; ^2^ Division of Training and Movement Sciences, Research Focus Cognitive Sciences, University of Potsdam, Potsdam, Germany; ^3^ Department of Orthopaedic Surgery and Traumatology, Faculty of Medicine, Suez Canal University, Ismailia, Egypt; ^4^ Department of Family Medicine, American University of Beirut (AUB), Beirut, Lebanon; ^5^ Department of Physical Therapy, College of Applied Medical Sciences, Imam Abdulrahman Bin Faisal University, Dammam, Saudi Arabia

**Keywords:** muscle activation, hamstring muscles, latency, injury risk, physical fitness expertise

## Abstract

**Purpose:** To examine the effects of fatiguing isometric contractions on maximal eccentric strength and electromechanical delay (EMD) of the knee flexors in healthy young adults of different training status.

**Methods**: Seventy-five male participants (27.7 ± 5.0 years) were enrolled in this study and allocated to three experimental groups according to their training status: athletes (ATH, *n* = 25), physically active adults (ACT, *n* = 25), and sedentary participants (SED, *n* = 25). The fatigue protocol comprised intermittent isometric knee flexions (6-s contraction, 4-s rest) at 60% of the maximum voluntary contraction until failure. Pre- and post-fatigue, maximal eccentric knee flexor strength and EMDs of the biceps femoris, semimembranosus, and semitendinosus muscles were assessed during maximal eccentric knee flexor actions at 60, 180, and 300°/s angular velocity. An analysis of covariance was computed with baseline (unfatigued) data included as a covariate.

**Results:** Significant and large-sized main effects of group (*p* ≤ 0.017, 0.87 ≤ *d* ≤ 3.69) and/or angular velocity (*p* < 0.001, *d* = 1.81) were observed. *Post hoc* tests indicated that regardless of angular velocity, maximal eccentric knee flexor strength was lower and EMD was longer in SED compared with ATH and ACT (*p* ≤ 0.025, 0.76 ≤ *d* ≤ 1.82) and in ACT compared with ATH (*p* = ≤0.025, 0.76 ≤ *d* ≤ 1.82). Additionally, EMD at post-test was significantly longer at 300°/s compared with 60 and 180°/s (*p* < 0.001, 2.95 ≤ *d* ≤ 4.64) and at 180°/s compared with 60°/s (*p* < 0.001, *d* = 2.56), irrespective of training status.

**Conclusion:** The main outcomes revealed significantly higher maximal eccentric strength and shorter eccentric EMDs of knee flexors in individuals with higher training status (i.e., athletes) following fatiguing exercises. Therefore, higher training status is associated with better neuromuscular functioning (i.e., strength, EMD) of the hamstring muscles in fatigued condition. Future longitudinal studies are needed to substantiate the clinical relevance of these findings.

## Introduction

Electromechanical delay (EMD) represents the time delay between onset of myoelectric activity and the corresponding increase in force/torque (Zhou et al., 1995a). It is well known that EMD is an integral part of the muscle contraction process and associated with neuromuscular performance (Conchola et al., 2013; Hannah et al., 2014). Specifically, it has been shown that longer knee flexor EMD are associated with an increased anterior cruciate ligament (ACL) injury risk (Conchola et al., 2013; De Ste Croix et al., 2015b). In this context, Hannah et al. (2014) compared EMD of the knee flexors (biceps femoris, semitendinosus) with that of the knee extensors (rectus femoris, vastus lateralis and medialis) during maximal isometric knee flexions and extensions in untrained male participants. The authors showed that knee flexor EMD was approximately twice as long as knee extensor EMD (44.0 vs. 22.6 ms, respectively). Given that ACL injuries experienced during sudden actions (e.g., cutting maneuvers, jump landings) are estimated to occur within 50 ms after ground contact (Krosshaug et al., 2007), authors concluded that the disbalance between knee flexor and extensor EMD may compromise knee stability, making it more vulnerable to ACL injuries (Hannah et al., 2014).

Numerous mechanisms have been suggested to underpin EMD such as the excitation-contraction coupling and the propagation of action potentials through the muscle membrane (Boncompagni et al., 2006), the stretching of the series-elastic component (Cavanagh and Komi, 1979; Herda et al., 2013), and/or the muscle fiber type composition (Nilsson et al., 1977; Viitasalo and Komi, 1981). For instance, it has been shown that higher type II muscle fiber (i.e., fast-twitch) proportions are associated with shorter EMD values (Viitasalo and Komi, 1981; Zhou et al., 1995a). In addition, there is evidence that the passive element of the series-elastic component (e.g., aponeurosis and tendon) shows an explained variance of 47.5% of the EMD during plantar flexion actions when electrically evoked (Nordez et al., 2009).

Previous studies have addressed the effects of neuromuscular fatigue (De Ste Croix et al., 2015b), musculotendinous stiffness (Badier et al., 1999), sex (Zhou et al., 1995b; De Ste Croix et al., 2015a), and age (Zhou et al., 1995b; Conchola et al., 2013) on EMD of different lower and upper limb muscles. Several authors provided evidence that neuromuscular fatigue significantly increased EMD (Zhou et al., 1996; Howatson, 2010; Conchola et al., 2013; De Ste Croix et al., 2015b). For instance, Conchola et al. (2013) studied the effects of neuromuscular fatigue in the form of submaximal intermittent isometric muscle actions on EMD of the leg extensors and flexors in young and old participants. The authors observed a significant and fatigue-related increase in EMD, irrespective of age. Conchola et al. (2013) hypothesized that the observed changes could be due to altered series-elastic components. This could be due to an increase in compliance characteristics and laxity of the tissue and/or a decrease in tendon stiffness (Conchola et al., 2013). In this regard, the time taken to produce intrinsic muscle force (i.e., EMD) to overcome an external load would increase (Conchola et al., 2013). Other mechanisms responsible for the observed findings could be changes in the excitation-contraction coupling and the propagation of action potentials through the muscle membrane (Conchola et al., 2013). These mechanisms appear to be affected by neuromuscular fatigue (Conchola et al., 2013). In addition, De Ste Croix et al. (2015b) examined the effects of soccer-specific fatigue on knee flexor EMD in young females of different age groups (i.e., U13, U15, and U17 years old) and reported significant fatigue-related increases in EMD (58.4%), irrespective of the age group.

Further, previous studies reported that higher muscle strength levels (Bell and Jacobs, 1986; Zhou et al., 1995a), higher musculotendinous stiffness (Zhou et al., 1995a), and faster muscle fiber conduction velocities (Viitasalo and Komi, 1981; Conchola et al., 2013) are associated with shorter EMDs. Of note, it is well documented that higher training status (e.g., endurance trained athlete vs. untrained person) is associated with higher fitness levels (e.g., maximal voluntary strength) and better fatigue resistance (Bachasson et al., 2016). Consequently, it could be argued that EMD is shorter in individuals with higher training status, particularly in fatigued conditions. In fact, Mitchell et al. (2011) examined differences in leg extensor/flexor EMD between power-trained (i.e., gymnasts), endurance-trained (i.e., swimmers), and untrained pre- and early pubertal boys. The authors demonstrated significantly shorter leg extensor EMDs in power-trained compared with untrained boys. However, to the best of our knowledge, the role of training status following neuromuscular fatigue on EMD has not been examined in a cross-sectional study yet.

While ample evidence is available on the effects of fatigue on leg-extensor EMD (Zhou et al., 1995b, 1996), only limited information has been reported on the effects of fatigue on EMD of the leg flexors (Conchola et al., 2013). More specifically, given the well-established function of the knee flexors during eccentric actions to stabilize the knee joint and preventing lower limb injury (e.g., ACL ruptures) (Bennell et al., 1998), it is crucial to examine EMD of the knee flexors during eccentric actions (Mesfar and Shirazi-Adl, 2006). Thus, future studies are needed that examine knee flexor EMD during eccentric muscle actions.

Given the above, this study aimed at examining the effects of neuromuscular fatigue on knee flexor EMD during the performance of eccentric muscle actions in healthy young adults according to their training status (i.e., athletes vs. physically active vs. sedentary). Based on previous literature (Conchola et al., 2015; De Ste Croix et al., 2015b; Bachasson et al., 2016), we hypothesized that neuromuscular fatigue induces longer knee flexor EMDs, particularly in individuals with low training status (Mitchell et al., 2011). Findings from this study could be clinically relevant for monitoring injury risk and for the implementation of injury preventive programs (Ristanis et al., 2009; Hannah et al., 2014; Flevas et al., 2017).

## Materials and Methods

### Participants

The local institutional and ethical review board approved this study prior to its start. Seventy-five healthy, male participants without any history of musculo-ligamentous complaints or any acute pain sessions in the knee joint were included in this study. Health status was assessed using a standardized health questionnaire (Physical Activity Readiness Questionnaire). Individuals (*N* = 75) were divided into three experimental groups (25 participants per group) according to their training status. Group 1 comprised athletes (ATH), group 2 included physically active adults (ACT), and group 3 comprised sedentary individuals (SED). Participants in the athletic group adhered to the following criteria: training in a specific sport stetting with the goal to improve sporting performance, regular participation in regional and national competitions, and officially licensed with a sport federation (Araujo and Scharhag, 2016). Participants of the active group were involved in recreational, leisure-time physical activities (e.g., walking, running, dancing, swimming, yoga, aerobics class, and playing on a sports team) (U.S. Department of Health and Human Services, 2018). Participants of the sedentary group were not engaged in any type of physical activities (i.e., sedentary lifestyle behavior). All participants signed an informed consent to participate in the current study and received an information sheet with detailed information on the experimental protocol. All experiments were conducted in accordance with the latest version of the declaration of Helsinki.

### Study Design

This is a comparative, cross-sectional study that aimed at examining the effects of neuromuscular fatigue on knee flexor EMD during the performance of eccentric muscle actions in healthy young adults of different training status (i.e., ATH, ACT, and SED). Participants visited the laboratory on three different occasions. The first two visits were dedicated to get participants accustomed to the experimental procedures (e.g., dynamometry) and the fatigue protocol. The third visit was devoted to the experimental trials during which the participants performed baseline tests followed by the fatigue protocol and post-tests in fatigued condition. Tests included the assessment of maximal eccentric knee extensor torque and myoelectric activity of the dominant leg. Leg dominance was determined according to the lateral preference inventory (Coren, 1993). Participants were instructed to refrain from caffeine uptake during the experiment as well as the day before the experiment. Additionally, they were instructed not to perform any strenuous physical exercises 48 h prior to the testing day.

### Assessment of Maximal Eccentric Knee Flexor Strength

**Figure 1:**
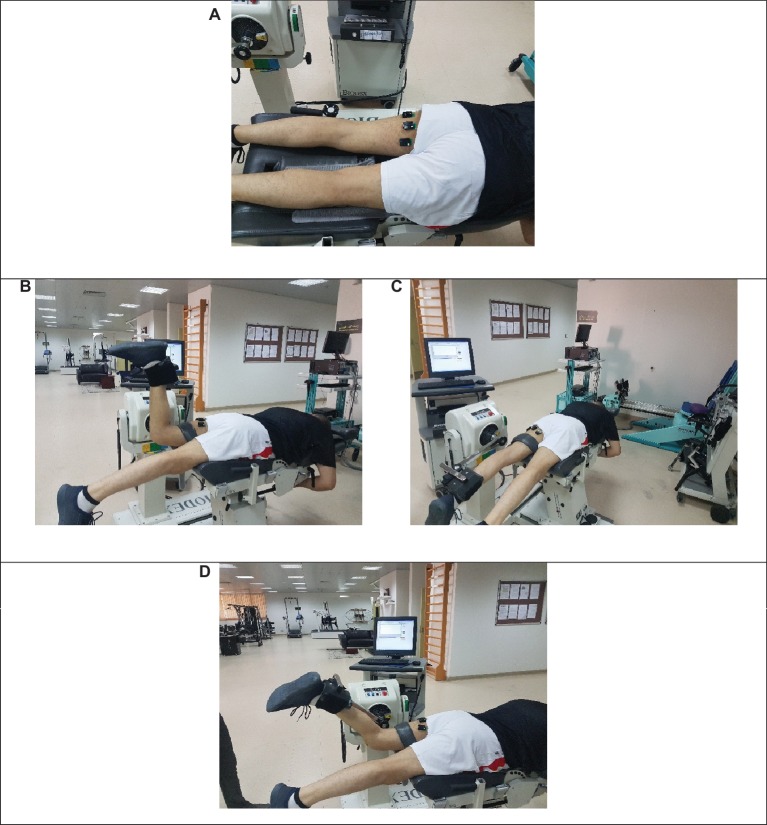
Testing procedures showing the zero-offset of myoelectric activity prior to testing **(A)**, the range of motion from 90° knee flexion **(B)** to full knee extension **(C)** during maximal unilateral eccentric knee flexor actions, and **(D)** knee flexion angle at 60° during the isometric fatigue protocol.

Maximal unilateral eccentric knee flexor actions were conducted with the dominant leg using a Biodex isokinetic dynamometer (Biodex, Shirley, NY, USA). During testing, participants were fixed on the dynamometer in prone position with the hip passively flexed at 10–20° (De Ste Croix et al., 2015a,b). The dominant leg was firmly attached to the lever arm of the dynamometer with its rotational axis corresponding to the level of the lateral femoral epicondyle. The brake pad was located about 3 cm superior to the medial malleolus with the foot in a relaxed state. In order to limit upper body contribution to torque production, straps/pads were applied at the hip and knee level. The range of movement was set at 90° knee flexion (initial position) to 0° (whereas 0° was defined as maximal knee extension) look Figures 1B,C. All settings were recorded during the practice session so that they were identical throughout the different experimental trials. Before testing, participants accomplished a 10-min standardized warm-up on a cycle ergometer (Monark Exercise, Vansbro, Sweden) at a self-selected intermediate intensity workload, with perceived exertion of 5–6 points on a 0–10 scale along with 5-min static and dynamic hamstring and quadriceps stretching. After the general warm-up, participants performed a specific warm-up on the isokinetic device consisting of three submaximal (self-perceived 50% effort) actions followed by two maximal concentric-eccentric knee extensions at 120°/s (El-Ashker et al., 2017). During pre- and post-tests, participants performed three maximal unilateral eccentric knee flexor actions per tested angular velocity (i.e., 60, 180, and 300°/s). The best trial was used for further statistical analyses. Following each eccentric muscle action, the examined leg was passively returned to the starting position (Prieske et al., 2017). The rest between the different angular velocities was 30 s. Participants were instructed to forcefully resist to the knee-extensor action which was produced by the dynamometer’s lever arm over the entire range-of-motion of the hamstrings (De Ste Croix et al., 2017; El-Ashker et al., 2019). At the beginning of each test session, participants were advised not to activate their muscles by staying fully relaxed. If the examiner noticed muscle preactivation in the myoelectric signal, participants were asked to relax before pushing the dynamometer lever arm (De Ste Croix et al., 2015a). Prior to each trial, participants were given verbal encouragement by the same examiner. All torque data were individually corrected for the effect of gravity using the dynamometer’s software prior to testing (Aagaard et al., 1998). Torque data were recorded at a sampling frequency of 1,000 Hz using an A/D converter (Delsys Myomonitor III, Delsys Inc., Natick, MA, USA) and low-pass filtered at 10 Hz (fourth-order, zero-lag Butterworth).

### Assessment of Knee Flexor Activity

During the assessment of maximal eccentric knee flexors torque, a wireless 8-channel Delsys electromyography (EMG) telemetric system (Delsys Myomonitor III, Delsys Inc., Natick, MA, USA) was used to record myoelectric activity of the biceps femoris, semimembranosus, and semitendinosus muscles. The average value of the three EMG signals in each angular velocity was recorded for further analyses. Electrodes were placed on the muscle bellies according to the European recommendations for surface electromyography (Hermens et al., 2000). The skin at the selected points were shaved, grazed, and cleaned with alcohol to decrease inter-electrode impedance and to increase the signal-to-noise ratio. Both EMG and torque data were synchronously analog-to-digital converted and recorded at a sampling rate of 1,000 Hz on the same I/O board. The raw EMG signals were filtered using a 20–450 Hz band-pass filter. The signal was full-wave rectified, and smoothed with a 100-ms moving root-mean-square algorithm. Before testing, the dynamometer and the EMG device were calibrated according to their respective manufacturer’s instructions. Additionally, a zero-offset of EMG activity was established during a 10-s time interval while in a relaxed prone position state prior to testing. In order to determine EMD, the time delay between onset of myoelectric activity and torque generation during maximal eccentric knee flexor actions was calculated (Lacourpaille et al., 2013). More specifically, EMD was defined as the time interval (in ms) between the onset of myoelectric activity of the semitendinosus, semimembranosus, and biceps femoris muscles (i.e., increase of 15-μV deviation from the baseline), respectively, and the corresponding torque development (i.e., 9.6-Nm deviation above the baseline level) during maximal eccentric knee flexor actions (Zhou et al., 1995a).

### Fatigue Protocol

About 5–10 min following baseline assessment, look Figure 1A participants performed an isometric fatigue protocol with the dominant leg on the isokinetic dynamometer. The same positioning on the isokinetic device was used as during testing. However, the knee flexion angle during the isometric fatigue protocol was set at 60°, look Figure 1D. The isometric fatigue protocol consisted of multiple sets of unilateral isometric knee flexions at 60% of the maximal voluntary contraction (MVC), 6-s duration with 4-s inter-set rest until failure (Vøllestad, 1997; Conchola et al., 2013; El-Ashker et al., 2019). Failure was defined as the time point when the participant could not maintain the pre-defined torque level for the third time (Vøllestad, 1997; Bilodeau et al., 2001). MVC torque of the knee flexors was assessed prior to the fatigue protocol using two maximal isometric knee flexions for 6 s and 1 min rest between trials. Throughout the fatigue protocol, each participant was instructed to track their torque outputs by viewing the real-time torque-time curve relative to the target torque level on a computer monitor (Thompson et al., 2015). Immediately, after the completion of the fatiguing protocol, post-tests were conducted. Verbal encouragement was given throughout the fatiguing protocol as well as during maximal eccentric knee flexor actions (Kimura et al., 1999; Campenella et al., 2000).

### Statistical Analyses

Data were examined for normal distribution and skewness using the Shapiro–Wilk test. Due to significant baseline between-group differences and because of the different angular velocities, an analysis of covariance (ANCOVA) with the factors group (ATH, ACT, and SED) and angular velocity (60, 180, and 300°/s) was computed with baseline data as a covariate. *Post hoc* tests with the Bonferroni-adjusted α were calculated to identify the comparisons that reached statistical significance. Effect sizes (ESs) were determined by converting partial eta-squared to Cohen’s *d* using the following equation: ES = 2 × sqr(eta^2/(1 − eta^2)) (Cohen, 1988). According to Cohen (1988), ES can be classified as small (*d* < 0.5), medium (0.5 ≤ *d* < 0.8), and large (*d* ≥ 0.8). Descriptive data are presented as group mean values and standard deviations. More specifically, post-test data are illustrated as baseline-adjusted group mean values and standard deviations. The significance level was set at *p* < 0.05. Data were analyzed using SPSS for Windows (version 25.0, SPSS, Inc., Chicago, IL, USA).

## Results

Anthropometric characteristics of the three groups are shown in Table 1. Baseline data on maximal eccentric knee flexor strength and EMD according to group and angular velocity are presented in Table 2.

**Table 1:** Participants’ anthropometric characteristics.

	ATH (*n* = 25)	ACT (*n* = 25)	SED (*n* = 25)
Age (years)	28.7 ± 4.2	28.3 ± 5.2	26.3 ± 5.6
Stature (m)	1.76 ± 0.0	1.7 ± 0.0	1.7 ± 0.0
Body mass (kg)	69.5 ± 4.3	78.7 ± 5.9	92.2 ± 5.7

ATH, athletes; ACT, physically active; SED, sedentary.

**Table 2:** Maximal eccentric knee flexor strength and electromechanical delay at baseline and different angular velocities (60, 180, and 300°/s) in male athletes (ATH), active (ACT) and sedentary adults (SED).

	ATH		ACT		SED		Differences (95% CI)		
	M	SD	M	SD	M	SD	ATH vs. ACT	ATH vs. SED	ACT vs. SED
**Maximal eccentric knee flexor strength (Nm)**									
60°/s	200.3	19.3	155.7	32.1	132.0	17.1	44.6 (28.1 to 61.0)	68.2 (51.8 to 84.7)	23.7 (7.2 to 40.1)
180°/s	154.0	19.6	135.7	29.5	107.6	11.8	18.3 (3.3 to 33.2)	46.4 (31.4 to 61.3)	28.1 (13.2 to 43.1)
300°/s	114.4	17.2	82.9	26.9	67.0	10.4	31.5 (18.1 to 45.0)	44.5 (31.0 to 57.9)	13.0 (−0.5 to 26.4)
**Electromechanical delay (ms)**									
60°/s	24.1	2.7	26.5	3.5	26.5	3.5	−2.47 (−4.7 to −0.2)	−2.39 (−4.6 to −0.1)	0.08 (−2.2 to 2.3)
180°/s	39.4	9.9	41.7	9.2	47.4	10.8	−2.32 (−9.2 to 4.6)	−8.07 (−15.0 to −1.1)	−5.75 (−12.7 to 1.2)
300°/s	54.5	6.3	54.7	7.8	55.8	6.9	−0.23 (−5.1 to 4.6)	−1.32 (−6.2 to 3.5)	−1.09 (−6.0 to 3.77)

M, mean; SD, standard deviation.

**Figure 2:**
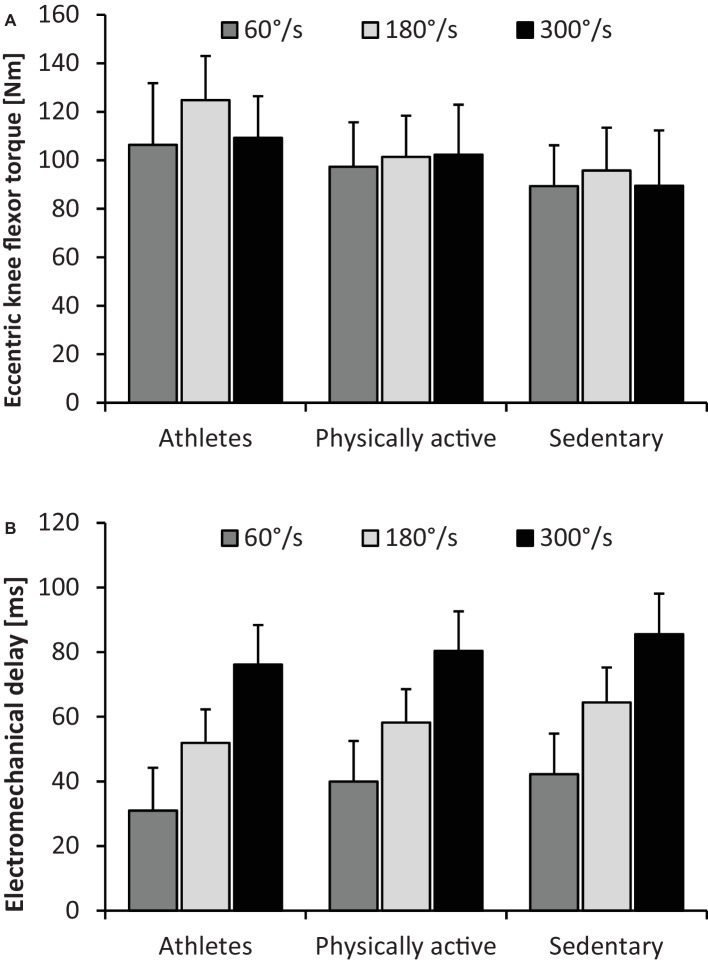
Means and standard deviations of maximal eccentric knee flexor strength [i.e., torque **(A)**] and electromechanical delay **(B)** in athletes, physically active and sedentary participants at different angular velocities during isokinetic testing (i.e., 60, 180, and 300°/s). Data were adjusted for baseline values.

Our statistical analyses revealed significant and large-sized main effects of group on maximal eccentric knee flexor strength and EMD (*p* ≤ 0.017, 0.87 ≤ *d* ≤ 3.69) and of velocity on EMD (*p* < 0.001, *d* = 1.81). *Post hoc* tests indicated higher maximal eccentric knee flexor strength at post-test in ATH compared with ACT and SED and in ACT compared with SED (*p* ≤ 0.010, 0.80 ≤ *d* ≤ 1.17), irrespective of angular velocity. Further, EMD at post-test was significantly longer in SED compared with ATH and ACT and in ACT compared with ATH (*p* ≤ 0.025, 0.76 ≤ *d* ≤ 1.82), irrespective of angular velocity. Additionally, EMD at post-test was significantly longer at 300°/s compared with 60 and 180°/s (*p* < 0.001, 2.95 ≤ *d* ≤ 4.64) and at 180°/s compared with 60°/s (*p* < 0.001, *d* = 2.56), irrespective of group. Baseline-adjusted post-test values are presented in Figure 2.

## Discussion

This study aimed at examining the effects of neuromuscular fatigue on knee flexor EMD during the performance of eccentric muscle actions in healthy young adults according to their training status (i.e., ATH vs. ACT vs. SED). The main results showed significantly higher maximal eccentric strength and shorter EMDs of knee flexors in individuals with higher training status (i.e., SED > ACT> ATH) following fatiguing exercises. Additionally, our findings indicated significant fatigue-related increases in EMD with increasing angular velocity (i.e., 300 > 180 > 60°/s). These findings suggest that a higher training status appears to be associated with a more efficient neuromuscular functioning (i.e., EMD) of the hamstring muscles in fatigued condition. These findings could be clinically relevant to identify persons at risk of sustaining ACL injuries (Hannah et al., 2014). However, further long-term training studies are needed to confirm the clinical relevance of our findings.

The present findings showed higher maximal eccentric knee flexor strength in ATH compared with ACT and SED and in ACT compared with SED irrespective of the angular velocity (i.e., 300 > 180 > 60°/s) following the fatiguing exercises. This observation indicates a training status-related fatigue resistance. The greater eccentric strength decrease following the fatiguing protocol in participants with lower compared to higher training status could compromise neuromuscular function and affect knee stability, amplifying the risk of ACL injuries (De Ste Croix et al., 2017).

It is well known that EMD is a clinically relevant marker of neuromuscular performance in different populations (Ristanis et al., 2009; Flevas et al., 2017; Norton and Eston, 2018). In this regard, short EMD reflects fast force transmission from the musculotendinous unit to the bone (Ricci Hannah et al., 2012). In general, longer EMD of hamstring muscles suggests impaired neuromuscular control which again affects knee joint stability (Hannah et al., 2014; Conchola et al., 2015). More specifically, there is evidence that short EMD of the hamstring muscles is crucial to protect the ACL from any excessive mechanical overload by providing stability to the tibia and by mitigating anterior tibia translation (Yanagawa et al., 2002; Hannah et al., 2014). In untrained male participants, Hannah et al. (2014) observed a delayed onset of knee flexor compared to extensor force production during the first 50 ms of maximal isometric contractions. According to the same authors, a longer knee flexor EMD impairs rapid force development of the knee flexors relative to the extensors which hampers knee stability and may leave the knee prone to ACL injuries during rapid muscle actions. Findings from this study showed that, irrespective of the angular velocity, EMD values in fatigued condition were longer in the SED compared to the ACT and ATH groups (0.76 ≤ *d* ≤ 1.82). Likewise, longer EMD values in fatigued condition were observed in ACT compared with ATH (*d* = 1.08). This might indicate that training status is associated with shorter EMDs, particularly in fatigued condition. There is evidence that fast hamstring muscle activation is important to protect the ACL from any excessive mechanical overload. With reference to our own data, low training status could be a risk factor for ACL injuries. This however needs to be substantiated in future longitudinal studies. Previous studies (Zhou et al., 1996; Mercer et al., 1998; Howatson, 2010; Conchola et al., 2013; De Ste Croix et al., 2015a,b) reported that fatigue-related increases in EMD are among others caused by the structural changes in series elastic components and tendon stiffness, the excitation-contraction coupling, and the propagation of action potentials through the muscle membrane.

To the authors’ knowledge, this is the first study that examined the effects of fatigue and training status on EMD in healthy young male adults. Previous studies were primarily conducted in youth (Mitchell et al., 2011). In fact, Mitchell et al. (2011) examined differences in EMD between power- (i.e., gymnasts) and endurance-trained (i.e., swimmers) athletes versus untrained subjects. However, potential fatigue-related effects were not addressed in this study. Mitchell and colleagues observed a significantly shorter EMD in power athletes compared with untrained subjects but not with endurance-trained athletes. These findings were shown for leg extensor but not flexor muscles. According to the same authors, the EMD difference between power-trained and untrained individuals was attributed to the higher musculotendinous stiffness in power athletes compared to endurance athletes and untrained individuals (Mitchell et al., 2011). De Ste Croix et al. (2015b) examined the effects of soccer-specific fatigue on EMD in female soccer players of various age groups (i.e., U13, U15, and U17). The authors demonstrated longer fatigue-related EMDs (Δ58%), irrespective of participants’ age and angular velocity. Specifically, they showed longer EMD in the younger (U13) compared with the older groups (U15 and U17). When translating these findings to ours, the longer EMD values in SED in fatigued condition compared to ACT and ATH as well as in ACT compared to ATH suggest that fatigue-related effects are more prominent in participants with low training status. This finding may indicate an increased risk of ACL injury in participants of low training status compared to athletes of high training status when controlled for exposition time. However, this needs to be verified in future studies.

In agreement with the literature (De Ste Croix et al., 2015a), our findings showed an increase in EMD with increasing angular velocity at post-fatigue. This means that neuromuscular function of the hamstring muscles is impaired at higher movement velocities. Accordingly, it can be speculated that knee joint stability is impaired at high angular velocity with a concomitant increase in injury risk (Hannah et al., 2014; De Ste Croix et al., 2017). De Ste Croix et al. (2015a) studied the changes in EMD of the hamstring muscles in males and females during eccentric muscle actions at different angular velocities (i.e., 60, 120, and 240°/s). The authors reported that, irrespective of sex, EMD increased with increasing angular velocity. Our results showed that, irrespective of the training status, increasing muscle contraction velocity could be associated with greater risk of ACL injury.

This study does have some limitations. First, the cross-sectional design of our study does not allow to establish cause-and-effect relations, which is why our findings have to be substantiated by future longitudinal studies. Second, the sporting activity distribution within each group was heterogeneous. This could have affected our findings as different EMDs are likely to be found in athletes from strength- (e.g., weight-lifting) versus endurance (e.g., triathlon)-dominated sports. Finally, the absence of reliability data of EMD may represent another limitation to this study. However, it has previously been demonstrated that EMD presents good reliability with coefficients of variation ranging from 3.1 to 6.5% depending on muscle action and movement velocity in physically active male subjects (Howatson et al., 2009).

## Conclusions and Future Perspectives

The main findings of this study showed higher maximal eccentric strength and shorter EMDs of the knee flexors in fatigued condition in young adults with higher compared to lower training status. These outcomes indicate that a higher training status could be associated with better neuromuscular functioning (i.e., EMD) of the hamstring muscles in fatigued condition. Longer fatigue-related EMDs in individuals with lower compared to higher training status could substantiate ACL injury risk. Accordingly, these findings could be of clinical relevance for the early detection of individuals with an increased risk of sustaining ACL injuries. However, this needs to be verified in future long-term training studies.

## Data Availability

All datasets generated for this study are included in the manuscript and/or the supplementary files.

## Ethics Statement

The local institutional and ethical review board of the Imam Abdulrahman Bin Faisal University, Dammam 31441, Saudi Arabia approved this study prior to its start. All participants signed an informed consent to participate in the current study and received an information sheet with detailed information on the experimental protocol. All experiments were conducted in accordance with the latest version of the declaration of Helsinki.

## Author Contributions

SE-A and HC conceived and designed the research. SE-A and AA conducted experiments. MA, OP, HC, and UG analyzed the data. SE-A, HC, OP, QM, and UG wrote the manuscript. All authors read and approved the manuscript.

### Conflict of Interest Statement

The authors declare that the research was conducted in the absence of any commercial or financial relationships that could be construed as a potential conflict of interest.
